# The OPTIMIZE study: protocol of a pragmatic sequential multiple assessment randomized trial of nonpharmacologic treatment for chronic, nonspecific low back pain

**DOI:** 10.1186/s12891-020-03324-z

**Published:** 2020-05-11

**Authors:** Richard L. Skolasky, Stephen T. Wegener, Rachel V. Aaron, Patti Ephraim, Gerard Brennan, Tom Greene, Elizabeth Lane, Kate Minick, Adam W. Hanley, Eric L. Garland, Julie M. Fritz

**Affiliations:** 1 Department of Orthopaedic Surgery, The Johns Hopkins University School of Medicine, 601 N. Caroline St, Baltimore, MD 21287 USA; 2 Department of Physical Medicine and Rehabilitation, The Johns Hopkins University School of Medicine, 601 N. Caroline St, Baltimore, MD 21287 USA; 3 Department of Epidemiology, The Johns Hopkins Bloomberg School of Public Health, 615 N. Wolfe St, Baltimore, MD 21205 USA; 4 Intermountain Healthcare, 36 S State St, Salt Lake City, UT 84111 USA; 5 Department of Population Health Sciences, University of Utah, 201 Presidents’ Cir, Salt Lake City, UT 84112 USA; 6 Department of Physical Therapy and Athletic Training, University of Utah, 201 Presidents’ Cir, Salt Lake City, UT 84112 USA; 7 College of Social Work, University of Utah, 201 Presidents’ Cir, Salt Lake City, UT 84112 USA

**Keywords:** Cognitive behavioral therapy, Comparative effectiveness research, Low back pain, Mindfulness, Physical therapy

## Abstract

**Background:**

Low back pain is a prevalent condition that causes a substantial health burden. Despite intensive and expensive clinical efforts, its prevalence is growing. Nonpharmacologic treatments are effective at improving pain-related outcomes; however, treatment effect sizes are often modest. Physical therapy (PT) and cognitive behavioral therapy (CBT) have the most consistent evidence of effectiveness. Growing evidence also supports mindfulness-based approaches. Discussions with providers and patients highlight the importance of discussing and trying options to find the treatment that works for them and determining what to do when initial treatment is not successful. Herein, we present the protocol for a study that will evaluate evidence-based, protocol-driven treatments using PT, CBT, or mindfulness to examine comparative effectiveness and optimal sequencing for patients with chronic low back pain.

**Methods:**

The Optimized Multidisciplinary Treatment Programs for Nonspecific Chronic Low Back Pain (OPTIMIZE) Study will be a multisite, comparative effectiveness trial using a sequential multiple assessment randomized trial design enrolling 945 individuals with chronic low back pain. The co-primary outcomes will be disability (measured using the Oswestry Disability Index) and pain intensity (measured using the Numerical Pain Rating Scale). After baseline assessment, participants will be randomly assigned to PT or CBT. At week 10, participants who have not experienced at least 50% improvement in disability will be randomized to cross-over phase-1 treatments (e.g., PT to CBT) or to Mindfulness-Oriented Recovery Enhancement (MORE). Treatment will consist of 8 weekly sessions. Long-term outcome assessments will be performed at weeks 26 and 52.

**Discussion:**

Results of this study may inform referring providers and patients about the most effective nonoperative treatment and/or sequence of nonoperative treatments to treat chronic low back pain.

**Trial registration:**

This study was prospectively registered on March 1, 2019, with Clinicaltrials.gov under the registration number NCT03859713 (https://clinicaltrials.gov/ct2/show/NCT03859713).

## Background

In the United States (U.S.), approximately 80% of adults experience at least 1 episode of low back pain during their lifetime, and 25% of adults report low back pain that lasted at least 1 day during the past 3 months [1]. Low back pain accounts for approximately 5% of all physician visits [2, 3] and is the third costliest health condition after diabetes and heart disease, with costs increasing at the second fastest rate of any health condition during the past decade [4]. Despite intensive clinical efforts, the prevalence of chronic low back pain continues to increase, affecting nearly 6% of U.S. adults at any given time [5]. Ineffective treatment of low back pain also contributes to the opioid crisis, given that low back pain is the most common diagnosis for which opioids are prescribed, despite a lack of evidence for their long-term benefits [5, 6].

Although acute low back pain often improves quickly, many individuals experience lingering or recurrent symptoms [7]. For those with chronic low back pain, approximately 30% report resolution of pain and disability after 1 year [8]. Evidence-based interventions for chronic low back pain exist. A recent review [9] identified 20 nonpharmacologic, noninvasive treatments with some level of supporting evidence. However, treatment effect sizes are modest, and individual patient responses are highly variable. Head-to-head comparisons of these modestly effective treatments typically result in equivocal findings, resulting in practice guidelines that consist of a list of possible treatments without direction about how to tailor or sequence treatments for an individual patient [10]. Patients with chronic low back pain report challenges to finding an effective treatment and deciding when to switch treatments when not achieving the desired results. In the absence of a universally effective treatment for chronic low back pain, research is needed to determine how to match patients with effective treatments, including whether specific sequences of treatments benefit distinct subgroups of patients [11].

Treatments with consistent evidence of effectiveness for chronic low back pain include physical therapy (PT) (exercise, education, and manual therapy) and cognitive behavioral therapy (CBT) (pain coping skills training, challenging negative thoughts, and relaxation training) [9]. In addition, a growing body of evidence supports mindfulness-based approaches for chronic pain [9, 12]. However, it is challenging for providers to predict which treatment will work for which patient, and whether certain treatment sequences are more effective than others. Traditional clinical trial designs are limited in their ability to examine these questions because patients are typically assigned to a fixed treatment regimen for the duration of the study, regardless of their response to treatment, and sample sizes are often insufficient to evaluate responses within patient subgroups [13]. Innovative trial designs are needed to examine treatment sequences and address intervention adaptations that should be made based on a patient’s responsiveness to care [14].

The Patient-Centered Outcomes Research Institute (PCORI) recognizes the burden of low back pain on individuals and society, as well as the difficulty that patients and referring providers experience in determining which is the right treatment at the right time for a given patient. In response, PCORI awarded a cooperative agreement to conduct a pragmatic clinical trial to determine the effectiveness of nonpharmacologic treatments to manage chronic low back pain, with a focus on determining the optimal sequencing of treatments. The design of this trial, entitled Optimized Multidisciplinary Treatment Programs for Nonspecific Chronic Low Back Pain (OPTIMIZE), is described herein.

## Methods/design

### Study design and rationale

**Fig. 1:**
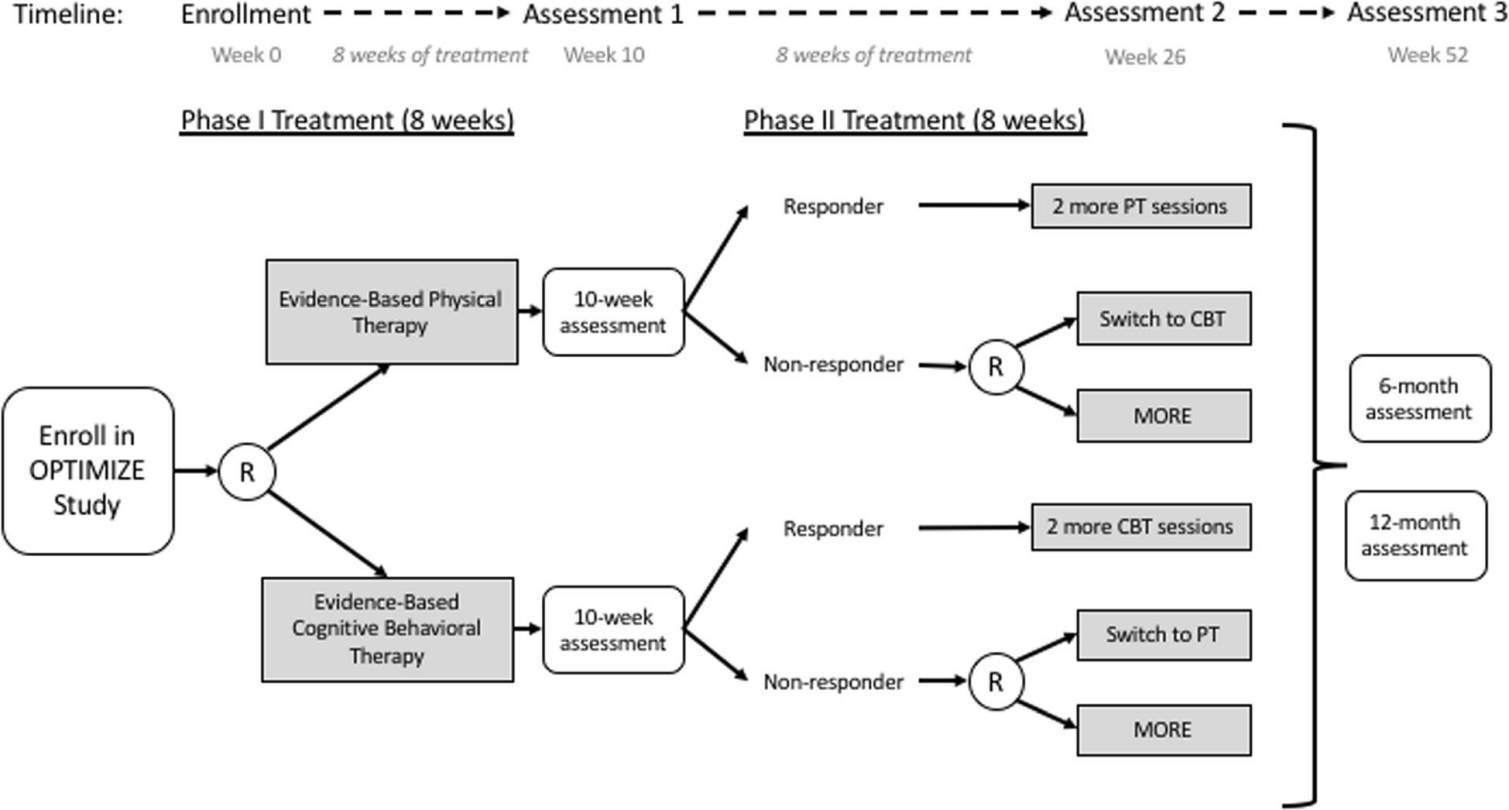
Intervention and assessment flow diagram for the OPTIMIZE Study, a sequential multiple assessment randomized trial. [Figure reprinted with permission.] CBT, cognitive behavioral therapy; MORE, Mindfulness-Oriented Recovery Enhancement; PT, physical therapy; R, randomize

The OPTIMIZE Study is a multisite comparative effectiveness trial using SMART (sequential multiple assessment randomized trial) design [15], with recruitment sites in 2 U.S. cities. SMART design allows for the assessment of adaptive interventions using prespecified decision rules to tailor treatment strategies to individual patients [16]. Figure 1 illustrates the study design. After informed consent and baseline assessment, participants will be randomized to receive 8 weekly sessions of phase-1 treatment with either PT or CBT. These treatments were selected on the basis of their common use [17, 18], supporting evidence [19], and lack of a previous head-to-head comparison in patients with chronic low back pain.

**Fig. 2:**
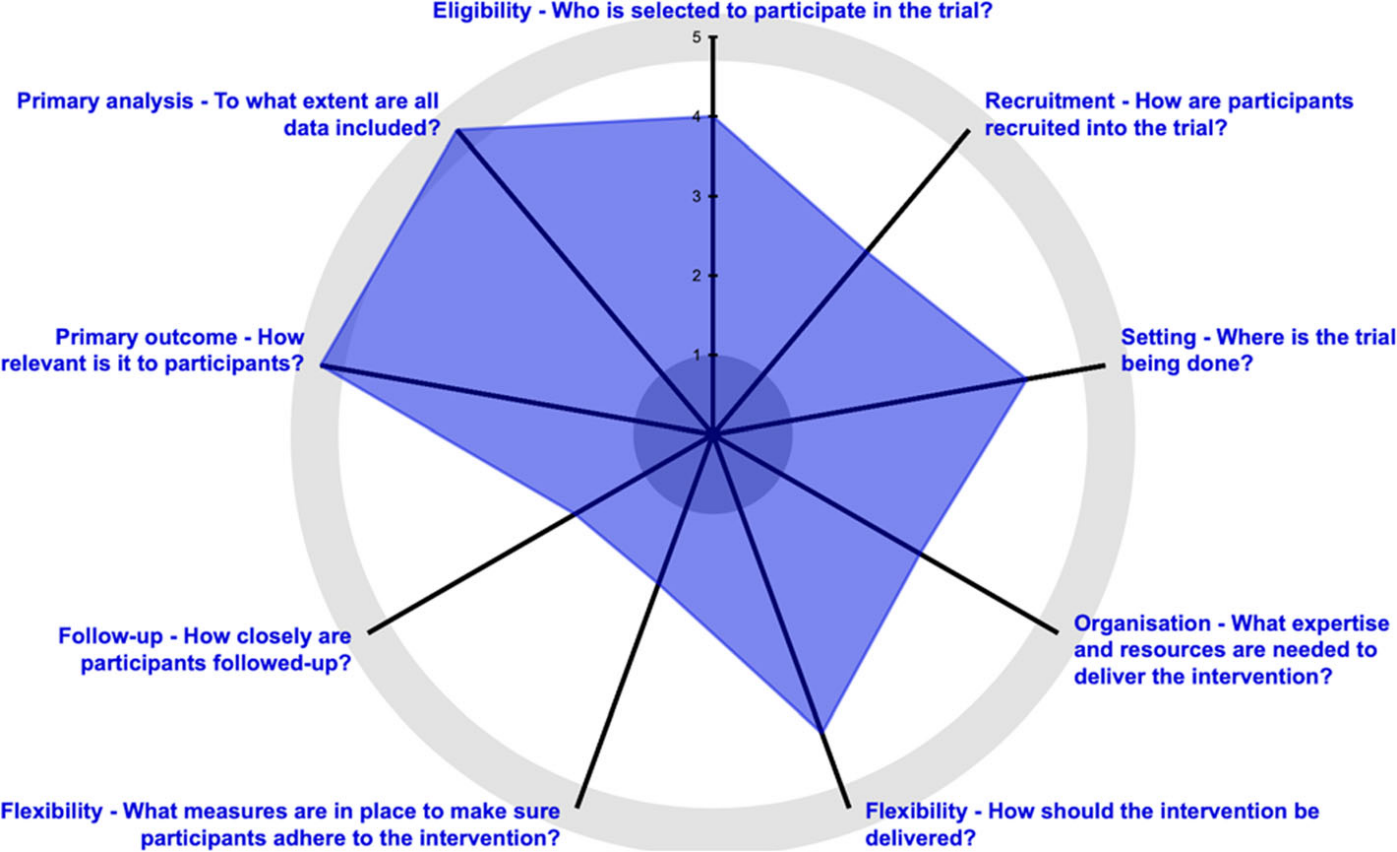
PRagmatic-Explanatory Continuum Indicator Summary-2 scoring wheel for the OPTIMIZE Study. Visual representation of pragmatism of the trial on the explanatory-pragmatic continuum. Scores of 1 to 5 on each spoke of the wheel indicate how pragmatic or explanatory the clinical trial is: 1, very explanatory; 2, rather explanatory; 3, equally pragmatic/explanatory; 4, rather pragmatic; and 5, very pragmatic. [Figure adapted with permission from Loudon K, Treweek S, Sullivan F, Donnan P, Thorpe KE, Zwarenstein M: The PRECIS-2 tool: designing trials that are fit for purpose. BMJ (Clinical research ed) 2015, 350:h2147]

Approximately 10 weeks after enrollment, participants who have not experienced adequate treatment response (i.e., 50% reduction in pain-related disability) to their assigned treatment will be randomly assigned to receive 8 weekly sessions of a new phase-2 treatment: 1) crossing over to the alternate phase-1 treatment (PT to CBT or CBT to PT) or 2) initiating Mindfulness-Oriented Recovery Enhancement (MORE). The rationale for the choice of phase-2 treatment options was based on a growing but less well developed evidence base and the existence of a provider workforce trained in mindfulness interventions in chronic low back pain. Switching treatments allows evaluation of the sequencing effects of PT and CBT. Participants who respond to phase-1 treatment are permitted up to 2 additional sessions during phase 2 to facilitate their transition to self-management. Outcome assessments are conducted at 10, 26, and 52 weeks after initial randomization. We scored the design of the OPTIMIZE Study using the 9 domains of the PRECIS-2 (pragmatic-explanatory continuum indicator summary-2) [20] on a scale of 1 to 5 and rated the study as more pragmatic than explanatory (Fig. 2 and Table 1).

**Table 1:** Application of PRagmatic Explanatory Continuum Indicator Summary (PRECIS)-2 Criteria to the OPTIMIZE Study

Domain	Criteria for scoring	Score	Rationale
Eligibility criteria	To what extent are the trial participants similar to those who would receive this intervention in usual care?	4	The eligibility criteria are similar to those that would be used in clinical decision making; assessments/screening are clinically available and routinely used
Recruitment path	How much extra effort is made to recruit participants than what is done in usual care settings to engage patients?	3	Recruiting from the electronic health record as a health system would to identify an at-risk population; use targeted invitation letters and incentives
Setting	How different are the resources, intervention provider expertise, and organization of care delivery in the trial from usual care?	4	Care is provided in the usual care settings; providers have been trained specifically for the study
Organization of intervention	How different are the settings for the trial from usual care settings?	3	Organization is identical to usual care; Back Pain Navigators serve a coordinating care role
Flexibility of experimental intervention–delivery	How different from usual care are the resources, intervention provider expertise, and organization of care delivery in the trial?	4	Allow flexibility per clinical judgement; there are intervention protocols, fidelity measurements, and engagement activities
Flexibility of experimental intervention–adherence	Is the intervention delivery in the trial more or less flexible compared with usual care?	2	Great effort is made to ensure that participants attend the first intervention appointment
Follow-up	How intense is the measurement and follow-up of trial participants compared with the typical follow-up of patients in usual care?	2	Assessments at baseline, week 10, and months 6 and 12 are outside of usual care; incentives are offered for completion
Outcome	To what extent is the trial’s primary outcome directly relevant to the participants?	5	Outcomes are highly relevant to participants and to providers
Analysis	To what extent will all data be included in the analysis of the primary outcome?	5	Intention-to-treat analysis is planned, using data from all randomized participants

The study design addresses 3 major deficits of previous investigations. First, given that many previous trials have shown small or equivocal effect sizes in terms of disability and pain, especially when averaged across study populations, our large sample size allows detection of smaller average treatment effects across the study population. Second, because of its large sample size, our study will have the statistical power to indicate whether particular treatment strategies are beneficial within subgroups based on specific patient characteristics. Third, the use of SMART design allows for the evaluation of sequences of treatments for participants who do not respond to the initial treatment.

### Ethical principles

Ethical review and approval were received from a local institutional review board (IRB), acting as the single IRB for this multisite study. Recruitment and local considerations were ceded to the individual site IRBs. Written consent was documented at 2 participating institutions and waived in lieu of verbal consent at 1 institution. Documentation of consent was obtained from all participants before study enrollment.

### Study objectives

The study comprises 3 broad objectives that each include a cluster of interrelated specific objectives.

#### Objective 1

At week 10, we will compare the effectiveness of phase-1 treatments (PT vs. CBT)

- in terms of primary outcomes, which are disability (using the Oswestry Disability Index [ODI]) and pain (using the Numerical Pain Rating Scale [NRS]);
- in terms of secondary outcomes, which are Patient Reported Outcome Measurement Information System (PROMIS) health domain scores and healthcare utilization measures (i.e., self-reported measures of seeking other types of care for chronic low back pain, such as chiropractic care, injections, or surgery); and
- by prespecified participant subgroups (defined by participant age, sex, opioid use, and psychosocial risk factors) in terms of ODI value and NRS score.

#### Objective 2

At 1 year, we will compare the effectiveness of phase-2 treatments (MORE vs. the alternate phase-1 treatment [herein, “switching”]) among nonresponders to phase-1 treatments in terms of

- ODI value and NRS score (separate comparisons for nonresponders to PT and nonresponders to CBT); and
- PROMIS health domain scores and healthcare utilization measures.

#### Objective 3

At 1 year, we will compare the effectiveness of phase-1 treatments (PT vs. CBT)

- in terms of ODI value and NRS score when MORE is used as the phase-2 treatment;
- in terms of ODI value and NRS score when switching is used as phase-2 treatment (i.e., PT followed by CBT or CBT followed by PT);
- in terms of PROMIS health domain scores and healthcare utilization measures when MORE is used as phase-2 treatment;
- in terms of PROMIS health domain scores and healthcare utilization measures when switching is used as phase-2 treatment;
- among prespecified patient subgroups when MORE is used as phase-2 treatment, in terms of ODI value and NRS score; and
- among prespecified patient subgroups when switching is used as phase-2 treatment, in terms of ODI value and NRS score.

### Study population

Eligibility criteria were designed to recruit a representative sample of patients with nonspecific chronic low back pain who recently sought healthcare for their condition. Reasons for ineligibility or nonparticipation will be monitored and recorded.

### Inclusion criteria

Eligibility criteria are as follows:

- able to speak English;
- aged 18–64 years;
- visited a healthcare professional for chronic low back pain during the past 90 days; and
- currently experiencing moderate pain and low back pain–related disability.

Chronic low back pain will be operationalized using the National Institutes of Health Task Force criteria based on 2 questions: 1) “How long has low back pain been an ongoing problem for you?” and 2) “How often has low back pain been an ongoing problem for you over the past 6 months?” Responses of “greater than 3 months” to question 1 and “at least half the days in the past 6 months” to question 2 will be required. Participant eligibility will be based on pain intensity scores of > 4 on the NRS and disability of ≥24% on the ODI.

### Exclusion criteria

Patients will be excluded from the study for the following reasons:

- serious pathology as a cause of low back pain, including neoplasm, inflammatory disease (e.g., ankylosing spondylitis), vertebral osteomyelitis, or other conditions;
- having received PT for low back pain during the previous 90 days;
- having received CBT or mindfulness therapy during the previous 90 days;
- having undergone lumbar spine surgery during the past year;
- current pregnancy; or
- current receipt of treatment or counseling for substance use (not including attending meetings of recovery programs such as Alcoholics Anonymous or Narcotics Anonymous).

### Study sites

OPTIMIZE will be conducted at 3 healthcare systems in the U.S.

### Recruitment, eligibility screening, and enrollment

Across study sites, potential participants will be informed of the study during healthcare visits with primary care providers. Alternatively, we will search the electronic health record (EHR) monthly to identify individuals with *International Classification of Diseases, Tenth Revision* codes indicating nonspecific low back pain (codes M47.817, M51.26, M51.27, M51.36, M51.37, M54.16, M54.17, M54.3, M54.4, M54.5, M54.89, M54.9, M99.03, M99.04, S23.9XXA, S33.5XXA, or S33.6XXA). Potential participants identified through the EHR will be sent an invitation letter via electronic or postal mail describing the study and providing instructions on how to opt out of additional contact or to opt in by contacting study personnel. Study personnel will attempt to contact by telephone those individuals who neither opt in nor opt out after 5 days.

Individuals interested in participation will be screened for eligibility using a telephone screener. Eligible individuals interested in participation will provide informed consent verbally or through a consent cover letter provided through Research Electronic Data Capture (REDCap), a Health Insurance Portability and Accountability Act–compliant, secure, electronic data capture system [21]. After provision of consent, participants will complete the baseline assessment through REDCap, supplemented by telephone assessment conducted by study personnel.

### Randomization and masking

The OPTIMIZE Study uses a SMART design involving 2 randomizations: one occurring after the baseline assessment and, for nonresponders, one occurring after the week-10 assessment. For each randomization, a computer-generated scheme will be used to randomize participants in a 1:1 ratio in blocks of random sizes stratified by enrollment site. Randomization will be administered centrally through the REDCap system [21].

It is impossible to mask treatment assignment from participants, healthcare providers, and study coordinators; however, study personnel who are responsible for baseline and follow-up assessments conducted through REDCap will be blinded to initial randomization assignment and re-randomization assignment, when applicable.

### Study measures

Assessments will be conducted at baseline (before randomization) and at weeks 10, 26, and 52 after enrollment (Fig. 1 and Table 2). The week-10 assessment corresponds to the end of phase-1 treatment, at which time, patients who did not respond to phase-1 treatment will be re-randomized. The week-26 assessment corresponds to the completion of phase-2 treatment. The 1-year assessment will permit evaluation of long-term treatment effects.

**Table 2:** Schedule of Research Assessments

Measure	Assessment Time Point				
	Pre-screening	Baseline	10 Weeks	6 Months	12 Months
Eligibility questions		X			
Informed consent form		X			
Baseline patient form		X			
Baseline researcher form		X			
Randomization		X	X^a^		
Follow-up researcher form			X		
Oswestry Disability Index	X^b^	X^b^	X	X	X
Numerical Pain Rating Scale	X^b^	X^b^	X	X	X
STarT Back Screening Tool		X	X	X	X
PROMIS domain					
Anxiety		X	X	X	X
Depression		X	X	X	X
Pain interference		X	X	X	X
Sleep disturbance		X	X	X	X
Social roles and activities		X	X	X	X
Treatment forms			X	X	
Adverse effects questionnaire			X	X	
Healthcare and opioid use		X	X	X	X

PROMIS, Patient Reported Outcome Measurement Information System

^a^Only if deemed “nonresponder” to phase-1 treatment

^b^To be administered at pre-screening assessment and repeated at baseline assessment only if > 14 days have passed since pre-screening

### Co-primary outcome measures

Our 2 co-primary outcome measures are low back pain–related disability and pain intensity.

#### Disability

The ODI is a 10-item measure of low back pain–related disability that assesses the current effects of a patient’s low back pain on various aspects of daily living. ODI scores range from 0 to 100, with higher scores indicating greater disability [22].

#### Pain intensity

Pain intensity will be assessed using an 11-item NRS ranging from 0 (“no pain”) to 10 (“worst imaginable pain”). Separate ratings will be recorded for current, best, and worst pain intensity during the previous 24 h [23].

#### Treatment response

The change in ODI value from baseline will be used to define a treatment responder. We previously evaluated ODI responder thresholds and found that patients who achieve at least 50% improvement are highly likely to consider themselves “a great deal” or “a very great deal better” [24]. We consider 50% or greater improvement in ODI from baseline to represent response to treatment.

### Secondary outcome measures

#### General health

We will use the PROMIS to assess physical, mental, and social health using the PROMIS-29 short form. The health domains assessed will be pain interference, physical function, fatigue, anxiety, depression, sleep disturbance, and ability to participate in social roles and activities (herein, “social roles and activities”) [25].

#### Long-term opioid use

We will ask participants to self-report opioid use at each assessment if they have used opioids for their low back pain during the past 90 days. For those responding “yes,” we will ask whether the patient has used opioids for their low back pain “daily or near daily in the past 90 days.” Daily or near daily use of opioids for at least 120 days is considered long-term opioid use [26].

#### Healthcare utilization

We will ask participants to self-report healthcare utilization at each assessment, including provider visits (e.g., primary care, complementary providers, emergency department, or surgical consults for low back pain), imaging (e.g., radiographs, magnetic resonance imaging), and procedures (e.g., injections, surgery).

### Additional measures

#### Demographic and clinical information

To characterize our participant sample, we will collect detailed demographic and clinical data at baseline (before randomization). Demographic data will consist of participant age, sex, race/ethnicity, employment status, and tobacco use and will be assessed through participant self-report. Clinical data will consist of general medical and low back pain history (e.g., previous back treatments and use of opioid medication). Demographic variables will be used to create patient subgroups and to determine possible confounding variables that may affect prognosis.

#### Adverse treatment effects

We will collect information about physical adverse effects (e.g., increased pain, stiffness) and psychological adverse effects (e.g., increased depression, anxiety) that participants report during week-10 and week-26 assessments. A questionnaire will ask participants whether they experienced any adverse effects and the extent to which they believe these adverse effects are related to study treatment (ranging from “not at all” to “extremely”) [27, 28].

#### Risk for poor outcome

We will assess each patient’s risk for poor outcome at baseline (before randomization) on the basis of psychological and physical risk factors using the STarTBack Screening Tool [29, 30] to characterize participants as having high, medium, or low risk for poor outcomes. Screening results from the STarTBack tool will be evaluated as a potential subgrouping variable.

### Implementation

In the setting of this pragmatic effectiveness trial, we will document barriers to implementation of study interventions to inform future implementation efforts. We will record barriers encountered across study sites using the Consolidated Framework for Implementation Research [31]. The Consolidated Framework for Implementation Research categorizes barriers into the following 5 domains: 1) characteristics of the intervention (e.g., aspects of the treatment); 2) outer setting (e.g., factors outside of the clinic and individual health systems); 3) inner setting (e.g., clinic environment); 4) providers (e.g., characteristics of the clinicians); and 5) process of intervention implementation. In addition to identifying and characterizing the barriers to implementation, we will record whether each barrier is ongoing or resolved and any actions taken to remedy the barrier.

### Treatments

All treatments will be provided by licensed providers who have at least 1 year of experience working with patients with chronic pain and who have been trained in study-related procedures by the investigators.

The initial treatment session will be provided within 30 days of randomization (to phase-1 or phase-2 treatment). All treatments will be provided during individualized weekly sessions over an 8-week period. To accommodate participants’ schedules, we will allow up to 2 sessions to be provided during the same week, but no more than 8 sessions will be received during the 8-week treatment period in phase 1 or phase 2. At the week-10 assessment, participants who are determined to have responded to phase-1 treatment may schedule 1 or 2 additional treatment sessions to finalize a self-management plan and discuss relapse prevention or other pertinent topics. The treatment approaches in this study are designed to be pragmatic, focusing on provision of evidence-based care but allowing tailored application of this care according to each patient’s needs.

### Treatment comparators

#### Physical therapy

Physical therapists will provide evidence-based PT for chronic low back pain consisting of patient education, exercise instruction, and manual therapy (Table 3) [9, 32]. Education will focus on reassurance, positive recovery expectations, addressing maladaptive pain perceptions, and the importance of physical activity [32] and may be tailored to individual patients’ needs based on the STarTBack Screening Tool [33]. Exercises will address general conditioning and physical activity, as well as deficits in strength, flexibility, and postural control tailored to the clinical presentation and needs of individual patients. Manual therapy may include various hands-on techniques tailored to the spinal mobility deficits of individual patients.

**Table 3:** Summary of evidence-based physical therapy

Session	Topics	Content and Patient Activities^a^
1	Assessment, establish exercise plan	Assess strength, flexibility, endurance deficits; develop exercise plan (minimum 20 min/day of home exercise); provide patient education and reassurance
2	SMT assessment, progress exercise	Identify spine mobility deficits; develop SMT plan and provide SMT; review exercise plan and progress (minimum 20 min/day of home exercise)
3	SMT, progress exercise	Provide SMT; review exercise plan and progress; increase daily home exercise to minimum 30 min.
4	SMT, progress exercise, review education	Provide SMT; review exercise plan and progress (minimum 30 min/day of home exercise); review patient education; elicit patient questions and concerns
5	SMT, progress exercise, self-management	Provide SMT; review exercise plan and progress (minimum 30 min/day of home exercise); discuss self-management plan
6	SMT, progress exercise	Provide SMT; review exercise plan and progress; increase daily home exercise to minimum 30 min
7	SMT, progress exercise, self-management	Provide SMT; review exercise plan and progress (minimum 30 min/day of home exercise); review self-management plan
8	Review and self-management	Finalize self-management plan and ongoing exercise program (minimum 30 min of home exercise 4–5 times/week); elicit and address patient questions and concerns

*SMT* Spinal manipulation therapy

^a^Each session includes reassessment and review of prior session and patient’s exercise and SMT program

#### Cognitive-behavioral therapy

CBT will be provided by behavioral health providers, including psychologists, advanced practice nurses, social workers, or other licensed providers with behavioral health training. The CBT protocol is adopted primarily from that of Thorn [34], as well as studies by Cherkin et al. [35] and Lamb et al. [36], showing effectiveness of CBT in patients with chronic low back pain. Patients will receive 8 sessions focused on key components of effective CBT (Table 4): 1) education about the biopsychosocial model of pain and its association with thoughts, feelings, and behavior 2) identifying and reframing maladaptive cognitions, 3) developing pain coping strategies (e.g., relaxation and positive coping statements), 4) setting and working toward behavioral goals using activity pacing, and 5) developing skills for self-management and relapse prevention [36, 37]. Depending on the judgment of the behavioral health provider, 2 additional CBT sessions may be offered to the patient to further address self-management and relapse prevention. Each course of CBT will begin with a psychosocial assessment (e.g., pain catastrophizing, fear of movement) and clinical interview. Patients will be instructed about activities to perform on their own between sessions and for ongoing self-management.

**Table 4:** Summary of evidence-based cognitive behavioral therapy

Session	Topics	Content and Patient Activities^a^
1	Assessment, stress and coping model of pain	Discuss attitude and beliefs about chronic pain and patient’s current approach to pain coping; identify relationships between stress, thoughts, feelings, behaviors, and physiology; learn relaxation exercise; complete assigned daily relaxation exercise and thought record
2	Behavioral activation	Learn techniques for activity pacing; develop graded activity plan; use goal-setting strategies to set specific physical and pleasant activity goals; assign goal setting activities.
3	Identifying negative automatic thoughts	Learn “gate control” theory of chronic pain; learn stress judging coping model of pain; learn types of negative thinking; identify negative thoughts and how they relate to thoughts, feelings, behaviors and physiology; assign daily thought record.
4	Changing negative automatic thoughts	Learn techniques for changing negative automatic thoughts to be more realistic; practice reframing negative automatic thoughts; assign daily thought record.
5	Changing core beliefs	Learn to identify “should” beliefs and core beliefs; learn techniques for changing core beliefs to be more realistic; practice reframing core beliefs; assign daily thought record.
6	Pain coping strategies	Create and use positive coping statements; practice passive muscle relaxation; assign regular positive coping statements and passive muscle relaxation at-home practice.
7	Effective communication	Learn and practice expressive writing; learn and practice assertive communication; assign regular at-home expressive writing and assertive communication.
8	Relapse prevention	Review skills learned in treatment; develop plan for using skills in future; assign ongoing practice of skills.

^a^Each session includes reassessment of patient’s beliefs and attitudes toward pain and review of prior session

#### Mindfulness-oriented recovery enhancement

MORE will also be provided by licensed behavioral health providers. The MORE program used in this study is designed specifically to address symptoms and underlying cognitive-affective mechanisms of chronic pain (Table 5) [38]. MORE will be provided in 8 individual sessions emphasizing 3 core therapeutic approaches:

- *Mindfulness:* Participants will be guided during each session to 1) become aware of when their attention is being engaged by pain or aversive thoughts and feelings; 2) acknowledge and accept that this attentional engagement has occurred; and 3) disengage attention from pain and aversive experience and then shift and engage attention to neutral or pleasant sensations via the practice of mindful breathing. Patients will also be taught to deconstruct the experience of pain into its sensorial components, using mindfulness to shift from affective to sensory processing of pain sensations by interoceptively mapping the location, distribution, and temporal dynamics of each sensation [39, 40].
- *Cognitive reappraisal:* After cognitive restructuring is introduced in session 3, participants will be taught to use mindfulness to become aware of and decenter from negative automatic thoughts, challenge automatic thoughts, and generate new, more adaptive appraisals.
- *Savoring of positive experiences:* After savoring of pleasant experiences is introduced in session 4, participants will be guided to use mindfulness to become aware of, focus attention on, and appreciate day-to-day positive experiences, as well as the pleasant sensations, positive emotions, and sense of meaningfulness arising in response to those experiences.

**Table 5:** Summary of mindfulness-oriented recovery enhancement

Session	Topics	Content and Patient Activities^a^
1	Automatic reactivity to pain	Introduction to mindfulness and the relationship between nociception, pain, and emotional suffering; mindful breathing and body scan
2	Cognitive control through mindfulness	Automatic pain coping habits; awareness of automatic coping; instruction in mindfulness of automatic pilot; mindful breathing
3	Mindful awareness of pain and stress-related cues	Mindful reappraisal as means of coping with negative emotions, stigma; mindful breathing
4	Shifting attention from pain or stress-related cues	Savoring natural rewards; positive emotion regulation; mindful savoring practice
5	Reorientation of attention through mindful breathing	Mindfulness of negative pain coping (e.g., bed rest, reliance on medication) and contemplation of negative consequences; mindful breathing practice
6	Reappraisal of maladaptive thoughts	Relationship of the stress response to pain and negative coping; imaginal stress exposure; mindful breathing; body scan
7	Moving between mindful disengagement and adaptive reappraisal	Concepts of thought suppression, aversion, and attachment; exercise in the futility of thought suppression; mindful breathing and acceptance
8	Review	Review; discussion of maintaining mindfulness practice; finding meaning and purpose of life; development of mindful recovery plan; imaginal rehearsal of skill learning; mindful breathing.

^a^Each session includes meditation practice, review of prior session, instructions for practice between sessions

Each course of MORE will begin with a psychosocial assessment and clinical interview. Patients will be instructed about activities to perform on their own between sessions and for ongoing self-management.

### Treatment fidelity

The study team has developed several mechanisms to enhance treatment fidelity for these study interventions. Mechanisms include provider training, structured intervention manuals and resources, and ongoing monitoring through the use of fidelity checklists embedded in the EHR.

#### Provider training

Study investigators develop a rigorous training schedule and materials for those who are providing the study interventions. Providers receive 12 h of training in study procedures and are provided manuals and online resources outlining core components for each treatment group. Initial training is conducted during an 8-h in-person workshop emphasizing demonstration and practice. PT and behavioral health providers will receive ongoing training through quarterly 1-h telephone calls led by an intervention leadership team to review protocols, reinforce skills, and discuss clinical issues. The calls will be discipline specific (i.e., a separate series of calls for PT and for behavioral health).

#### Structured intervention manuals

For each of the 3 study interventions, the study team has developed structured intervention manuals that follow the 8-session format. The manuals offer guidance to healthcare providers and include informational handouts and worksheets for participants.

#### Fidelity assessment

Providers complete checklists built into the electronic medical record to document treatment sessions, providing a pragmatic assessment of treatment fidelity [41]. We will use these checklists to determine whether core components of each study intervention are provided to participants. After each treatment session, the provider will complete fidelity checklists through the EHR.

### Statistical design

All analyses will follow intention-to-treat principles, with participants evaluated on the basis of randomization assignment, regardless of compliance. Personnel at a biostatistics center at one of the enrollment sites will perform statistical analyses.

Our primary objectives are to compare the effectiveness of the phase-1 treatments and of the phase-2 treatments among phase-1 nonresponders. Our main secondary objective is to compare the effectiveness of each phase-1 treatment when followed by MORE or when followed by switching to the alternative phase-1 treatment. As exploratory objectives, we seek to identify which two-stage embedded treatment regime provides the best average outcome across the four 2-stage treatment strategies and to evaluate the primary and secondary treatment comparisons in prespecified patient subgroups.

#### Compare effectiveness of phase-1 treatments

Separate longitudinal linear models will be used to relate repeated assessments of our co-primary outcomes of disability and pain to phase-1 treatment (PT versus CBT), while controlling for baseline outcome score. Model parameters will be estimated using normality restricted maximum likelihood estimation [42]. With this approach, treatment effect estimates will remain consistent and unbiased if missing data follow a missing-at-random pattern. Mean differences in ODI values and NRS scores at week 10 will be our primary assessment. Secondary comparisons of PT and CBT at weeks 26 and 52 will evaluate long-term effects of phase-1 treatment in the context in which nonresponders are assigned to a phase-2 treatment with equal probability.

A similar analytic framework will be used to compare the effects of phase-1 treatments (PT versus CBT) on secondary outcomes (physical function, anxiety, depression, fatigue, sleep disturbance, and social roles and activities). Weighted generalized estimating equations will be used to compare participant opioid use during the 1-year follow-up period between those who underwent PT vs CBT as phase-1 treatment [43]. Differences at week 10 will again represent our primary assessment, with subsequent comparisons in the context of phase-2 treatment for nonresponders.

Subgroup analyses for phase-1 treatments will be performed by repeating the longitudinal analyses within each prespecified group and by adding interactions between phase-1 treatments and subgroup factors comprising age (< 50 or ≥ 50 years), gender, long-term opioid use (yes/no), and high risk according to the STarTBack screening tool.

#### Compare effectiveness of phase-2 treatments among phase-1 nonresponders

To determine which treatment to use when phase-1 treatments do not provide adequate response, we will compare the effectiveness of switching to MORE versus CBT for phase-1 nonresponders to PT, and, in a separate analysis, we will compare the effectiveness of switching to MORE versus PT for phase-1 nonresponders to CBT. We plan separate analyses for the nonresponders to PT and CBT because the characteristics of these nonresponders may differ. These comparisons will be performed in the 2 sets of nonresponders using separate longitudinal linear models for our co-primary outcomes at weeks 26 and 52, with the phase-1 treatment and week-10 outcome scores used as covariates to account for the phase-1 treatment and its initial effects before implementing the phase-2 treatment.

Similar analysis will be conducted to determine the effectiveness in terms of secondary outcomes of phase-2 treatments among phase-1 nonresponders.

A preplanned secondary analysis will pool the estimates of the effects of phase-2 treatments across the 2 groups of phase-1 nonresponders. This analysis will receive particular emphasis if the overall study encounters a shortfall of recruitment or if treatment adherence is low, which would limit the statistical power of the primary comparisons performed separately in the nonresponders to PT and CBT.

#### Compare effectiveness of phase-1 treatments when followed by MORE

We will compare the effectiveness of the 2 phase-1 treatments when followed by MORE in analyses that include all responders to the phase-1 treatments, as well as nonresponders who are randomized to MORE. By using weighted generalized estimating equations, we will compare disability and pain at weeks 10, 26, and 52 between the 2 phase-1 treatments. Inverse probability weights will account for nonresponders being re-randomized into 2 groups (switching vs. MORE) and, therefore, being underrepresented relative to responders to phase-1 treatment. Similar methods will be used to determine the effectiveness of phase-1 treatments followed by MORE in terms of secondary outcomes and within prespecified subgroups.

#### Compare effectiveness of phase-1 treatments when followed by switching

We will compare the effectiveness of the 2 sequences of the phase-1 treatments (i.e., CBT followed by PT vs. PT followed by CBT) by using analyses analogous to those described above for the comparison of the phase-1 treatments when followed by MORE. Analyses will include responders to the phase-1 treatments and nonresponders who were randomized to the alternate phase-1 treatment in phase 2.

### Multiple comparison adjustment

Analyses addressing objectives 1 and 3 will use 2-sided α levels of 0.04 for the ODI and 0.01 for the NRS to assure a study-wide type-I error ≤ 0.05 across the 2 co-primary outcomes. Because the comparisons for objective 2 will be applied separately among phase-1 nonresponders to CBT and PT, we will use 2-sided α levels of 0.02 for the ODI and 0.005 for the NRS. This will ensure that the total type-I error for the objective 2 comparisons will not exceed α = 0.05 = 0.02 (for the ODI in CBT nonresponders) + 0.02 (for the ODI in PT nonresponders) + 0.005 (for the NRS in CBT nonresponders) + 0.005 (for the NRS in PT nonresponders). We will use different α levels for the 2 co-primary outcomes because the minimum clinically important difference for the NRS is larger in relation to its variability than it is for the ODI. (i.e., NRS scores are less variable because we expect to observe a larger difference in relation to its underlying variability than the difference we expect to observe in ODI values.)

### Sensitivity analyses

Treatment will be assigned randomly at the participant level and provided by trained physical therapists, psychologists, and social workers. Although the statistical analyses described above will be performed at the participant level, we realize that there may be an effect of provider on treatment effect. To account for this, we will conduct planned sensitivity analyses within each broad objective to test for effects of clustering. First, we will evaluate whether treatment effects vary across sites. We will use a random effects model to test treatment effects in our primary and secondary objectives that include main effect for site and interaction effects between site and treatment group. Second, we will evaluate whether treatment effects vary across providers. Using a random effects model, we will add random effect terms for the participants’ index providers, recognizing that each participant may have more than 1 intervention provider.

### Statistical power

Statistical power was evaluated under the assumption of an estimated 85% participant retention during the 1-year follow-up period. We have designated ODI value and NRS score as our co-primary outcomes and assume standard deviations of 12.5 and 2.2, respectively. We assume serial correlations of 0.13 and 0.23 for the ODI and NRS, respectively, between baseline and follow-up according to a previous study [44]. The computations assume phase-1 responder rates of 30% to 45% [24, 45] and account for the type-I error rates described above. Each study intervention is an active treatment hypothesized to be beneficial; therefore, we base our power calculations on minimal clinically important differences in ODI (6 points) and NRS (2 points) [45, 46] instead of directional hypotheses for comparisons against control groups. Under the indicated assumptions, 945 randomized participants provide at least 99% power for the objective-1 and objective-3 comparisons and at least 89% power for the objective-2 comparisons when performed in the full randomized cohort. All of these comparisons have sufficient power to determine whether one treatment is clinically superior to the other or whether the mean difference between treatments is sufficiently small that the treatments can be considered clinically equivalent. The objective-1 and objective-3 comparisons retain at least 83% power when performed in subgroups that include at least one-third of the randomized participants.

### Safety monitoring

The risks of the interventions are minimal because PT, CBT, and MORE are standard treatments used in everyday clinical practice. Through our study eligibility criteria (e.g., excluding those with serious pathology as cause of low back pain) and other procedures (e.g., requiring licensed providers to deliver interventions), we have attempted to minimize risks to participants. All investigators and research staff complete online tutorials and in-person training approved by their institutional review boards to comply with all regulations of the Office of Human Research Subjects Protection. A data and safety monitoring board composed of individuals with relevant expertise from outside the participating institutions will provide external safety monitoring for the OPTIMIZE Study.

Participant safety and confidentiality will be monitored continuously. Treatment providers, through training and ongoing conference calls, will report any potential adverse events to the site study coordinators. In addition, adverse events will be solicited at every assessment and, if they occur, will be recorded and reported in accordance with standard reporting guidelines of our respective institutional review boards and PCORI and our data and safety monitoring plan. The relatedness, expectedness, and severity of adverse events are adjudicated by the study investigators and data and safety monitoring board.

## Discussion

Many clinical trials examining nonpharmacologic, noninvasive treatments for chronic low back pain have been published [9, 12]. Although several treatments are supported by this body of evidence, effect sizes are small, and direct comparisons typically find little difference between effective interventions [9, 47]. It is unlikely that any single intervention will prove to be universally beneficial for patients with chronic low back pain. Therefore, advancing care for such patients requires new research designs beyond parallel-group, fixed intervention trials. Clinical trials are needed to address questions of how to adapt and sequence interventions when initial treatment efforts fail [48]. Because the diagnosis of chronic low back pain comprises heterogeneous causes and symptoms, clinical trials need large sample sizes to allow rigorous evaluation of the tailoring of interventions according to prespecified patient characteristics that may influence effectiveness [47, 49, 50]. The OPTIMIZE Study is designed to address these key considerations.

Although PT and CBT are among the most common treatments for chronic low back pain and have the most robust evidence supporting their effectiveness [9], we are aware of no previous clinical trial comparing efficacy of these treatments directly. Because the OPTIMIZE Study uses PT and CBT as phase-1 interventions, we will be able to compare the effectiveness of these interventions and do so with sufficient sample size for subgroup analyses in the context of SMART design. The study design also allows us to investigate whether the sequence of these 2 interventions influences their effectiveness. Because PT and CBT are commonly available to patients with chronic low back pain, the question of which treatment to offer first could affect care pathways in healthcare systems. Mindfulness is an evidence-based intervention for chronic pain that is attracting increased interest among patients [50]. We decided to use the MORE intervention as a phase-2 treatment because it is a recently developed mindfulness-based intervention that has not been widely integrated into healthcare systems [51] but has been shown in two stage-2 randomized controlled trials to significantly reduce the severity of chronic pain, the degree to which pain interferes with daily life, and the misuse of prescription opioids [52, 53]. Using mindfulness as a phase-2 treatment allows comparisons with PT and CBT among phase-1 nonresponders and analysis of the sequencing effects of preceding mindfulness with either PT or CBT.

Several limitations to our study warrant discussion. Although SMART design has several advantages, an important aspect of this approach is designating a single variable to define “response” to treatment. Although evidence supports the validity and relevance to patients of our threshold of 50% improvement in low back pain–related disability [24], some participants’ perceptions of their treatment response may not be accurately reflected by this threshold. Our SMART design includes 2 treatment phases; however, our conversations with patients indicate that some try many more treatments before finding one that is effective. Several other nonpharmacologic treatments have evidence of effectiveness for chronic low back pain, including acupuncture and massage, but these are not included in our study. Despite these limitations, the OPTIMIZE Study will provide foundational evidence on which to build a cost-effective adaptive treatment strategy for population-level chronic low back pain intervention.

The OPTIMIZE Study will help patients with chronic low back pain and their healthcare providers identify treatments that will be effective for them. This study is the largest trial to investigate the effectiveness of PT, CBT, and MORE and the first to investigate the effects of different sequencing of these treatments. The long-term goal is to give patients and providers the information needed to select the interventions that are most likely to lead to better health outcomes and, if the first attempted treatment is ineffective, to optimize the sequencing of treatments to reduce disability, alleviate pain, and improve quality of life.

## Data Availability

Not applicable.

## Abbreviations

CBT: Cognitive behavioral therapy
MORE: Mindfulness-Oriented Recovery Enhancement
NRS: Numerical Pain Rating Scale
ODI: Oswestry Disability Index
OPTIMIZE: Optimized Multidisciplinary Treatment Programs for Nonspecific Chronic Low Back Pain
PCORI: Patient-Centered Outcomes Research Institute
PROMIS: Patient Reported Outcome Measurement Information System
PT: Physical therapy
SMART: Sequential multiple assessment randomized trial
